# Human papillomavirus-associated anal squamous intraepithelial lesions in men who have sex with men and transgender women living with and without HIV in Karachi Pakistan: implications for screening and prevention

**DOI:** 10.1186/s12879-021-06850-w

**Published:** 2021-11-17

**Authors:** Muslima Ejaz, Muhammad Mubarak, Tazeen Saeed Ali, Sören Andersson, Anna Mia Ekström

**Affiliations:** 1 Department of Global Public Health, Global and Sexual Health Research Group, Karolinska Institutet, Widerströmska Huset, Tomtebodavägen 18A, 171 77 Stockholm, Sweden; 2 Department of Community Health Sciences, Aga Khan University (AKU), Stadium Road, P.O. Box 3500, Karachi, 74800 Pakistan; 3 Department of Cytology and Histopathology, Sindh Institute of Urology and Transplantation, Karachi, Pakistan; 4 School of Nursing, The Aga Khan University, Karachi, Pakistan; 5 Department of Laboratory Medicine, Faculty of Medicine and Health, Örebro University, Örebro, Sweden

**Keywords:** Anal cytology, Anal cancer, ASIL, LSIL, HSIL, HPV infection, Anal Pap smear, HIV, Men who have sex with men, MSM, STI, Transgender, Pakistan

## Abstract

**Background:**

Anal squamous intraepithelial lesions (ASIL), strongly related to human papilloma virus (HPV) infection, is more prevalent among men who have sex with men (MSM). However, no such data are available for Pakistan yet, and neither HPV vaccination nor anal-cytology screening is implemented in Pakistan. The purpose of this first ever study was to assess the prevalence of HPV-related anal cytological abnormalities among MSM and transgender women living with and without HIV infection in Pakistan.

**Methods:**

We conducted a cross-sectional study from March 2016 to November 2017 at sexual health centers run by the Perwaaz Trust and the National AIDS Control Program in Karachi. The study enrolled MSM and transgender women aged greater-than-and-equal-to-18-years who reported anal sex in the preceding 6 months. We collected two anal samples for liquid-based cytology and HPV type testing by PCR, and socio-demographic and behavioral data were collected through face-to face interviews. ASIL and its associations with biological and behavioral risk factors were analyzed through Cox regression for prevalence ratios (PR) and corresponding 95% confidence intervals (CIs).

**Results:**

Out of 271 qualifying participants, 79% were MSM and 21% transgender women. The mean age was 28.8 (± 8) years. Almost 35% (93/271) of the study population had ASIL detected, ASIL was significantly more common among participants living with HIV than in HIV negative ((50/118) 42.4%; vs. (43/153) 28.1%) (p ≤ 0.001). Among ASIL, 66% (61/93) had low-grade squamous intraepithelial lesions (LSIL), and 3.6% (3/93) had high-grade squamous intraepithelial lesions (HSIL). The overall, HPV16 positivity was 35.5% (33/93) among all abnormal anal lesions and all 3 HSIL were HPV16 positive, however, HPV16 positivity could show its association with ASIL detection in univariate model only (PR_crude_: 2.11(1.39–3.18)). Moreover, any HR-HPV type (PR 3.04; 95% CI 1.75–5.26), concurrent sexually transmitted infection (STI) (2.13; (1.28–3.55)) and HIV + /HPV + coinfection (1.75; (1.07–2.88)) remained independently associated with ASIL in the multivariate model.

**Conclusions:**

Abnormal anal cytology among MSM and transgender is prevalent enough to consider optimal screening regimens. Further studies are required to see if periodic anal cytology can be made part of HIV care and treatment programs among MSM in Pakistan.

**Supplementary Information:**

The online version contains supplementary material available at 10.1186/s12879-021-06850-w.

## Background

Persistent infection with Human Papillomavirus (HPV)—predominantly high-risk HPV type 16 is the major cause of anal cancer [1] Over the past decade, the global incidence of anal squamous cell carcinoma (SCC), is rare and stable in the general population [2] however, it is 20 times more likely to occur in men who have sex with men (MSM) [3, 4] compared to heterosexual men [5]. The risk of anal cancer is highest in MSM living with HIV [4, 6–8], other individuals living with HIV [9], immunosuppressed individuals [10], and women with history of HPV associated anogenital dysplasia [11]. The reported incidence of anal cancer in general population is 1.8 per 100,000 persons [12], in MSM it ranges between 19–35 cases per 100,000 and up to 88–131/100,000 in MSM living with HIV, i.e., more than 70 times more common than in the general population [9, 13–15]. The increasing incidence of anal cancer can be associated with increases in the number of sexual partners and a higher prevalence of anal sex in the past few decades [16, 17].

Anal squamous intraepithelial lesions (ASIL) are the putative precursors of anal cancer, similar to cervical squamous intraepithelial lesions (CSILs) in cervical cancer [18–22]. ASIL spans from low grade squamous intraepithelial lesions (LSIL) to high grade squamous intraepithelial lesions (HSIL) [23]. Approximately 90% of LSIL are caused by infection with low-risk HPV types 6 and 11 often clinically presenting as condyloma—genital warts—while infections with high-risk HPV types 16 and 18 are associated with HSIL [24]. However, persistent anal infection with both low-risk or high-risk HPVs and related ASILs have been associated with an increased risk for invasive anal cancer [24, 25]. Both people living with HIV and MSM generally have a higher prevalence and incidence of anal canal and perianal HSIL [21, 26]. The impact of antiretroviral therapy (ART) for HIV appears to be very limited in terms of reducing the risk of HSIL or squamous cell cancer of the anal canal and perianal region [27, 28].

Although there is a dearth of evidence-based uniform screening recommendations as well as benefits verses harm appraisals [29], however, recent research suggests that anal Papanicolaou (Pap) screening may have utility in preventing anal cancer [30–32], and is cost-effective screening method for anal cancer prevention among MSM [30, 33] Anal cytology can be used as a standard screening tool for individuals at risk for anal cancer to detect early stage ASIL as screening tests used for cervical cytology [24, 34, 35]. High levels of abnormal cytology have consistently been reported among previously unscreened MSM both without (12–32%) and with (34–58%) HIV [36–38] in various settings.

Anal cytology screening and preventive treatment programs for MSM have not yet been routinely adopted by Pakistan for multiple reasons, including an absence of local prevalence estimates, sufficient clinical expertise and infrastructure and updated guidelines for the management of sexually transmitted infections (STI). To contribute evidence for future policy, prevention, screening and management of the anal cancer burden in Pakistan, we studied HPV, ASIL, STIs and associated socio-demographic, behavioral and clinical factors among MSM and transgender women with and without HIV infection in Karachi, Pakistan.

## Methods

### Study design and study setting

A cross-sectional study was conducted from March 2016 to November 2017 among MSM and transgender women aged ≥ 18 years who reported having had anal sex in the preceding 6 months. Using snowball and peer referral sampling techniques, participants were recruited from a male sexual health clinic run by community-based organization Perwaaz Trust in Karachi, Pakistan. The Perwaaz Trust provides information and screening for STI, HIV testing and counseling and other health care services to MSM. We also recruited MSM and transgender women with known HIV infection from a public sector HIV and ART center run by the National AIDS Control Program of the Government of Pakistan, situated at the Civil Hospital in Karachi. This is the largest (1900-bed) tertiary care public hospital in Karachi and is a large public research and teaching hospital attached to the Dow University of Health Sciences, Karachi. Consecutive MSM and transgender women living with HIV who were attending their quarterly visits at the center between March 2016 to November 2017 were invited to participate. The study was approved by the Human Research Ethics Committee of The Aga Khan University (3612-CHS-ERC-15) and Dow University of Health Sciences Karachi Pakistan (IRB-557/DUHS/APPROVAL/2015/84).

### Study procedure

After explaining the purpose of the study and study procedures, potential participants were asked to provide written informed consent. Thereafter, they were invited to participate in a face-to-face interview and to provide anal samples to detect the presence of HPV as well as any cytological lesions in the perianal area and the anal canal. A blood sample was obtained for confirmation of their HIV status (through Architect HIV Ag–Ab Combo kit by ABBOTT), viral load and CD4 + T-cell count (through FACS Count TM flow cytometry, Becton Dickinson Co., Franklin Lakes, NJ, USA). Participants were informed that they were free to interrupt the interview or withdraw from the study at any time without any negative consequences in terms of clinical services provided to them.

### Data collection

A validated structured questionnaire with 45 questions was administered by the principal investigator (ME) and a trained data collector in a confidential space to collect information on participants’ socio-demographic characteristics, sexual behavior (the past 6 months), reproductive and medical history, HIV history, any anal disease and any use of ART. The interview lasted for an average of 35–40 min. To establish the validity of the questionnaire, pilot testing was done on 10% of the sample size. A local panel of experts established content validity. Internal consistency of the questions was established by Cronbach’s alpha wherein values between 0.70 and 0.90 were obtained. Data were coded by the principal investigator (ME) and entered by two independent data operators. Data were validated and corrected for any disparity. Frequencies were run on data for logical error checking and for any discrepancy, and study participants who had given their consent to be contacted were contacted through telephone or asked when visiting the study settings for correction of any errors.

### Biological sample collection

In order to exclude the presence of any ano-genital warts, all participants underwent an inspection of their external genitalia and perianal area by a trained physician who also collected an anal sample using a moistened sterile Dacron swab (Copan Diagnostics Inc, Corona, CA, USA). The swab was inserted 3–5 cm into the anus without any direct visualization and then rotated against the anal canal wall for a minute before being removed slowly in a spiral fashion. Immediately after sampling, the swab was inserted into 3 mL of a methanol-based fixative, a sample transport medium (Universal Transport medium, Corona CA, USA), and transferred to a pathology laboratory for cytological assessment by two experienced cytopathologists at the Sindh Institute of Urology and Transplantation. About 0.5 ml of the cell suspension from the collected sample was drawn with an adjuster and put into a disposable Shandon EZ mega funnel for smear preparation in a cytospin. Glass slides were labelled and fixed into the megafunnel. The sample was centrifuged at 1000 rpm for 10 min. After 10 min, the slides were removed from the cytospin, fixed in alcohol and stained with both hematoxylin and eosin (HE) stain and Papanicolaou stain. The anal cytology was classified using the Bethesda criteria for cervical cytology [39]. Abnormal cytology was classified as atypical squamous or epithelial cell abnormalities, including atypical squamous cells of undetermined significance (ASCUS), LSIL, atypical squamous cells that do not exclude high-grade lesions (ASC-H) and HSIL [39].

HPV-testing was performed on anal PreservCyt^®^ specimens using Polymerase Chain Reaction (PCR) for detection of low-risk (6/11) and high-risk (group 1) genotypes (16/18/31/33/35/39/45/52/56/58/59).

### Statistical analysis and study variables

In total, 298 men and transgender women were recruited, out of which 27 (9.0%) were excluded from the cytological analyses due to insufficient biological material and thus omitted from the statistical analyses as well. Data analyses were performed using SPSS version 24.0 (Statistical Package for Social Sciences TX, USA). The prevalence of ASIL and 95% confidence intervals (CI) were calculated using the exact binomial method. The associations between biological and behavioral risk factors (independent variables) and cytologically identified ASIL (dependent variable) were analyzed using Cox regression hazard model algorithms with robust SEs to estimate prevalence ratios (PRs) and corresponding 95% CI. Biological determinants included HIV status; anal STIs, i.e., gonorrhoea (*Neisseria gonorrhoeae*), trichomonas and anal warts diagnosed or reported in the last 6 months; and anal HPV infection as determined by PCR. Anal symptoms including anal bleeding, itching or any pus discharge were also considered. Behavioral factors included number of sexual partners, consistent (always) condom use, smoking status (yes/no, if yes age at initiation; average number of cigarettes per day < 10, between 10 and 20, > 10; and were cigarettes filtered or unfiltered) and drinking alcohol (yes/no, if yes how many times; < 5 times, between 5 and 10, or more than 10 times/week) were also analyzed for their association with the dependent variable ASIL. A *t*-test was also performed to compare the mean number of types of HPV infections between those with and without ASIL.

## Results

This study included a total of 118 participants living with HIV and 153 without HIV in the final cytological and statistical analyses. Those living with HIV were significantly older than HIV negative participants (30.9 versus 27.2; *p* ≤ 0.001), among PLWH 89.3% were on combination ART, 74.8% had recent CD4 count of more than 350 cells/μl) and nearly two-thirds (76.3%) had undetectable HIV viral load at their last assessment. There were also significant differences in the duration of sex work, number of sexual partners, receptive anal intercourse, and inconsistent condom use at anal intercourse in the last 6 months between study participants living with versus without HIV (Table 1).

**Table 1:** Socio-demographic and lifestyle characteristics of MSM and Transgender women living with and without HIV also stratified on HIV Status

Characteristics	Study population (N = 271)	HIV positive (N = 118)	HIV negative (N = 153)	p-value
Mean (± SD)	28.8 (± 8.06)	30.9 (± 0.70)	27.0 (± 0.59)	< 0.001
Age categories in years n (% ages)				
< 25	90 (33.2)	23 (19.5)	67 (43.8)	< 0.001
25–29	84 (31.0)	38 (32.2)	46 (30.1)	
30–34	36 (13.3)	19 (16.1)	17 (11.1)	
≥ 35	61 (22.5)	38 (32.2)	23 (15.0)	
Marital status n (% ages)				0.001
Unmarried	211 (77.9)	79 (66.9)	132 (86.4)	
Married	53 (19.6)	34 (28.8)	19 (12.4)	
Separated/divorced	7 (2.5)	05 (4.3)	02 (1.2)	
Smoking history				
Current status: n (% ages)				
Yes	210 (77.5)	99 (83.9)	111 (72.5)	0.02
Age at smoking initiation in years mean (± SD)	16.5 (± 4.9)	15.5 (± 4.2)	18.0 (± 95.7)	< 0.001
Average number of cigarettes/day n (% ages)				
< 10	118 (56.2)	50 (50.0)	68 (64.8)	0.02
10–20	65 (31.0)	34 (34.0)	31 (29.5)	
> 20	27 (12.8)	16 (16.0)	6(5.7)	
Duration of smoking in years Mean (± SD)	12.4 (± 8.5)	14.83 (± 9.0)	10.15 (± 7.7)	< 0.001
< 5 years n (%)	60 (28.6)	16 (16.0)	44 (39.6)	< 0.001
5–10 years n (%)	51 (24.3)	21 (21.0)	30 (27.0)	
11–15 years n (%)	64 (30.5)	39 (39.0)	26 (23.4)	
> 15 years n (%)	35 (16.6)	24 (24.0)	11 (9.9)	
Sexual behaviors				
Sexual identity n (% ages)				
Bisexual	58 (21.4)	34 (29.8)	24 (17.4)	0.008
Homosexual	156 (57.6)	55 (45.0)	101 (64.7)	
Transgender	57 (21.0)	33 (25.2)	24 (17.9)	
Sex worker characteristics n (%)	215 (79.3)	91 (77.1)	124 (81.0)	0.43
Age first had sexual intercourse (in years) mean (± SD)	16.42 (± 4.7)	15.13 (± 3.8)	17.44 (± 5.1)	< 0.001
Duration of sex-work in-years mean (± SD)	12.42 (8.2)	15.76 (8.3)	9.8 (7.1)	< 0.001
Anal receptive sexual partners the last 6 months n (% ages)*				
< 25	23 (8.5)	4 (3.6)	19 (13.9)	< 0.001
25–50	31 (11.4)	4 (3.6)	27 (19.7)	
51–100	83 (30.6)	33 (30.0)	50 (36.5)	
> 100	110 (40.6)	69 (62.7)	41 (29.9)	
Condom use during anal receptive sex n (%)				
Consistent use	60 (22.1)	12(10.2)	48 (31.4)	
Inconsistent use	163 (60.1)	95 (80.5)	68 (44.4)	< 0.001
Never use	48 (17.7)	11 (9.3)	37 (24.2)	
Preferred role at anal sex				
Mainly receptive	243 (89.7)	113 (95.8)	130 (85.02)	
Mainly insertive	28 (10.3)	5 (4.2)	23 (15.0)	0.004
Presence of other STI	160 (59.0)	88 (74.6)	72 (47.1)	< 0.001
History of anal warts during previous 6 months				
Yes	205 (68.8)	104 (79.4)	101 (60.5)	< 0.001

*Almost 80% of the study population was invloved in sex work. These categories of sexual partners in last six months i.e., (< 25), (25–50), (51–100), (> 100). Greater number by and-large reflects their "clients"

HIV status (53.8% vs 38.1%; p = 0.014), age at sex work initiation (15.6 vs 17.0 years p = 0.02), inconsistent condom use (74.7% vs 52.3% p = 0.001) and receptive anal sex (95.8% vs 86.4% p = 0.01) were associated with greater prevalence of ASIL detection (Table 2).

**Table 2:** Socio-demographic and lifestyle characteristics of MSM and transgender women living with and without HIV in Karachi, Pakistan, by Anal Squamous Intraepithelial Lesions (ASIL) status

Characteristics	ASIL positive (N = 93)	ASIL negative (N = 178)	p-value
Age in years mean (± SD)	28.2 (± 7.8)	29.2 (± 7.8)	0.31
Age in categories n (% age)			
< 25	37 (39.8)	53 (26.8)	0.24
25–29	28 (30.1)	56 (31.5)	
30–34	08 (8.6)	28 (15.7)	
≥ 35	20 (21.5)	41 (23.0)	
Education n (% age)			
None	33 (35.1)	48 (27.0)	0.113
Middle school or less	27 (28.70)	41 (23.0)	
High school or less	27 (29.8)	77 (43.2)	
Graduate	6 (6.4)	12 (6.8)	
Marital status n (% age)			
Unmarried	74 (79.5)	137 (77.0)	0.924
Married	17 (18.3)	36 (20.2)	
Separated/divorced	02 (2.2)	05 (2.8)	
Smoking status n (% age)			0.302
No	18 (18.9)	43 (24.4)	0.07
Yes	77 (81.1)	133 (75.6)	
Age at smoking initiation in years; mean (± SD)	15.74 (± 4.2)	16.90 (± 4.2)	
Average number of cigarettes/day n (% age)			
< 10 10–20 > 20	39 (54.2) 25 (34.7) 08 (11.1)	79 (59.4) 40 (30.1) 14 (10.5)	0.758
Duration of smoking in years mean (± SD)	12.0 (± 8.6)	12.5 (± 8.6)	0.68
Alcohol drinking n (% age)			
Yes No	27 (28.4) 68 (71.6)	34 (19.3) 142 (80.7)	0.087
HIV status: n (% age)			
Yes No	50 (53.8) 43 (46.2)	68 (38.1) 110(61.8)	0.014
Sexual identity: n (% age)			
Bisexual Homosexual Transgender	17 (18.3) 46 (49.5) 30(32.2)	43 (24.4) 102 (57.3) 33 (18.5)	0.047
Age first had sexual intercourse			
In years mean (± SD)	15.6 (± 4.4)	17.05 (± 4.8)	0.02
Duration of sex work in years mean (± SD)	12.5 (± 8.2)	12.15 (± 7.9)	0.70
Anal receptive sexual partners the last 6 months n (% age)			
< 25 25–50 51–100 > 100	9 (10.2) 6 (15.7) 26 (35.8) 47 (39.6)	14 (8.8) 25 (15.7) 57 (35.8) 63 (39.6)	0.079
Condom use during anal receptive sex n(% age)			
Consistent use Inconsistent use Never	8 (8.4) 71 (74.7) 16 (16.8)	52 (29.5) 92 (52.3) 32 (18.2)	< 0.001
Preferred anal sex role n (% age)			
Mainly receptive Mainly insertive	91 (95.8) 4 (4.2)	152 (86.4) 24 (13.6)	0.01*
History of anal warts in the previous 6 months n (% age)			
Yes No	71 (74.7) 24 (25.3)	118 (67.0) 66 (33.0)	0.188

*Fisher Exact test p-value

### Cytologic assessment

**Fig. 1:**
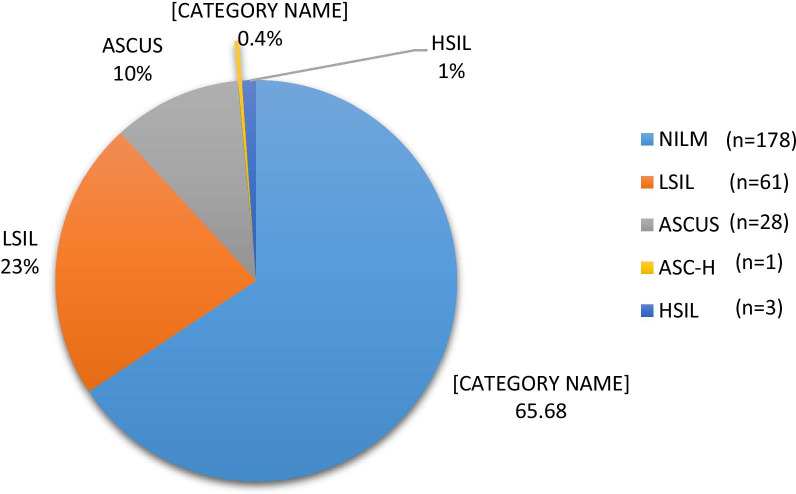
Distribution of anal squamous intraepithelial lesions (ASIL) among MSM and Transgender women. *NILM* negative for intraepithelial lesions or malignancy, *ASCUS *atypical squamous cells of undetermined significance, *LSIL* low grade squamous intraepithelial lesions, *ASC-H* atypical squamous cells do not exclude high-grade lesion, *HSILs* high grade squamous intraepithelial lesions

**Fig. 2:**
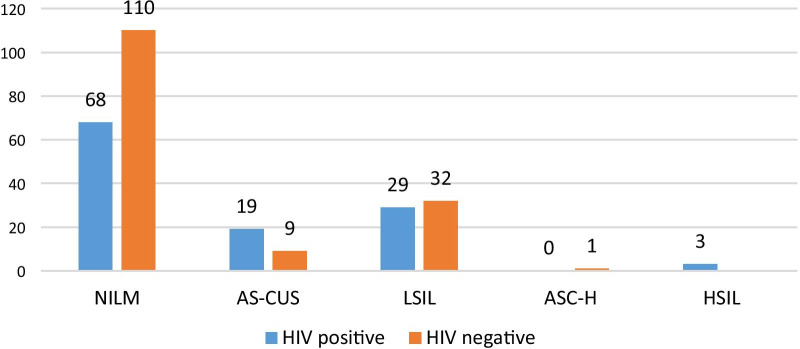
Distribution of anal squamous intraepithelial lesions (ASIL) stratified on HIV status among MSM and transgender women. *NILM* negative for intraepithelial lesions or malignancy, *ASCUS *atypical squamous cells of undetermined significance, *LSIL* low grade squamous intraepithelial lesions, *ASC-H* atypical squamous cells do not exclude high-grade lesion, *HSILs* high grade squamous intraepithelial lesions

Twenty-seven participants (9%) were excluded due to inadequate sample cellularity making them insufficient for cytologic assessment, leaving 271 participants (HIV positive n = 118; HIV negative n = 153) for the cytological and statistical analyses. Out of 271, a total of 93 (35.0%) MSM and transgender women had any ASIL (ASCUS, LSIL, ASC-H or HSIL) (Fig. 1). Participants living with HIV were significantly more likely to have such an abnormality as compared to those who were HIV negative ((50/118) 42.4%; vs. (43/153) 28.1%) (p ≤ 0.001); (Fig. 2). Age was not associated with the detection of cytological abnormalities (*p* = 0.31). Just one HIV-negative participant had ASC-H (who had multiple HPV types: LR-HPV 6 & 11 and HR-HPV 35) while three (2.5%; 3/118) participants living with HIV had developed HSIL and all three samples of HSIL were positive for HPV16. HIV infected participants were infected with multiple HPV types than HIV uninfected participants (p < 0.001) (Table 3).

**Table 3:** Multiplicity of HPV types among any anal squamous intraepithelial lesions (ASIL) and HIV status among MSM and transgender women in Karachi Pakistan

# of types n (%)	All N = 271	ASIL positive n = 93	ASIL negative n = 178	p-value	HIV positive n = 118	HIV negative n = 153	p-value
None	92 (33.9)	0 (13.0)	92 (52.3)	< 0.001*	15 (12.7)	77 (50.3)	< 0.001
One Type	64 (25.1)	32 (40.0)	32 (17.0)		27 (22.9)	23 (15.1)	
Two types	84 (29.5)	40 (37.9)	44 (25.0)		47 (39.8)	35 (22.9)	
Three types	27 (10.0)	20(21.1)	07 (4.0)		24 (20.3)	12 (7.8)	
Four types	4 (1.5)	1 (1.1)	3 (1.7)		5 (4.3)	6 (3.9)	

p-value < 0.001 *Fisher exact test p value

### The overall and type specific HPV infection

Overall HPV infection prevalence among study population was 66.4% (180/271). Study participants living with HIV were significantly more infected with HPV (60%; 108/180) than participants living without HIV (40%; 72/180) (p value ≤ 0.001). Among 180 HPV positive participants, 83% (n = 149/180) had at least one HR-HPV type among them. Overall HPV16-positivity among all HPV positive was 35% (n = 63/180).

**Fig. 3:**
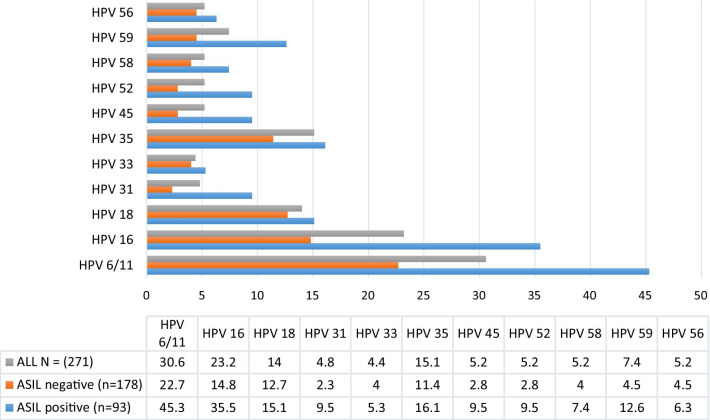
Distribution of HPV genotypes (in percentages) stratified on anal squmous intraepithelial lesions (ASIL) status among MSM and transgender women

All participants with ASIL (n = 93) were HPV positive. Within ASIL, the most prevalent HPV types were HPV6/11 (39.8% (n = 37)) followed by HPV16 (35.5% (n = 33), HPV35 (16.1%; n = 15) and HPV18 (15%; n = 14) (Fig. 3) (Additional file 1: Table S1).

### Abnormal anal cytology associated risk factors

The detection of ASIL was associated with type of HPV infection, in particular HPV16 (PR_crude_ = 2.11, 95% CI 1.39–3.18), known as one of the high-risk types for cancer development, while HPV type 35 was the second most common oncogenic type among study participants and was associated with 59% increased prevalence of ASIL detection (PR_crude_ = 1.59, 95% CI 0.99–2.58). Participants living with HIV were more likely to have such an abnormality as compared to those who were HIV negative (PR_crude_ = 2.33, 95% CI 1.36–2.41). However, all these factors could not appear as an independent risk factor for the greater prevalence of ASIL detection in the multivariate model.

Nevertheless, having any HR-HPV type (16/18/31/33/35/45/52/58) (PR 3.04; 95% CI 1.75–5.26), having another concurrent STI (PR; 2.13, 95% CI 1.28–3.55) and being co-infected with both HPV and HIV (PR 1.75; 95% CI 1.07–2.88) were independently associated with greater prevalence of ASIL detection while adjusting for all other behavioral and sexual variables in the multivariate model (Table 4).

**Table 4:** Factors associated with any abnormal anal cytology (n = 93) among MSM and transgender women living with (n = 118) and without HIV 9n = 153) in Karachi Pakistan

Determinants	Any abnormal anal cytology Crude PR (95% CI)	p values	Any abnormal anal cytology Adjusted PR (95% CI)
HIV status			
Negative (ref.)	1.0		
positive	2.33 (1.36–2.41)	< 0.001	
Any HR-HPV types			
No (ref.)	1.0		**1.0**
Yes	3.8 (2.25–6.51)	< 0.001	**3.04 (1.75–5.26)**
HPV type 16			
No (ref.)	1.0		
Yes	2.11 (1.39–3.18)	< 0.001	
HPV type 35			
No (ref.)	1.0		
Ye	1.59 (0.99–2.58)	0.060	
Other STI^a^			
No (ref.)	1.0		**1.0**
Yes	2.77 (1.68–4.59)	< 0.001	**2.13 (1.28–3.55)**
HIV/HPV co-infection			
No (ref.)	1.0		**1.0**
Yes	2.93 (1.92–4.45)	< 0.001	**1.75 (1.07–2.88)**
HIV/STI^a^ co-infection			
No (ref.)	1.0		
Yes	2.04 (1.36–3.04)	0.001	
Preferred anal sex role			
Insertive (ref.)	1.0		
Receptive anal sex	2.62 (0.96–7.13)	0.059	
Condom use			
Always (ref.)	1.0		
Inconsistent use	3.27 (1.57–6.78)	0.002	
Never use	2.50 (1.07–5.84)	0.034	
Number of sex partners in past 6 months^b^			
< 100 (ref.)	1.0		
≥ 100	1.43 (0.94–2.17)	0.096	

*PR *prevalence ratio

^a^STIs included: gonorrhea and trichomonas

^b^79.3% of our study participants were sex workers (Table 1)

## Discussion

This is the first study to report on the prevalence and risk factors of any anal abnormal cytology among MSM and transgender women in Pakistan. Not unexpectedly, we found a strong association between concurrent high-risk HPV, HIV infection and ASIL. The overall proportion of MSM and transgender women with ASIL was 35%, and those living with HIV were almost twice as likely to have an abnormal cytology compared with HIV negative participants (42.4% versus 28.1%). Moreover, and as expected, the prevalence and number of high-risk HPV types was higher among those with an HIV infection, including HPV16—the primary causal agent for anal cancer. The prevalence of estimates for ASIL in Pakistan are consistent with previous studies from the Asian region [40–42] and elsewhere [27, 34, 43–46]. However, the ASIL rates that we found among in MSM in Pakistan are slightly higher than what has been reported from India (27.7%) [47], naturally influenced by study population and sampling. To date, no South Asian studies among MSM (including ours) have used high resolution anoscopy for histologic confirmation of the ASIL diagnosis, which is considered to be the gold standard method [34]. Given that previous studies have shown that anal cytology is less sensitive when diagnosing ASIL compared to the gold standard anal biopsy with the histopathological results [34], the true prevalence of ASIL is likely to be higher than found in our study. Furthermore, and as a matter of concern, we found unexpectedly high proportions of anal cancer precursor lesions, i.e., ASC-US + (35.8%) among young HIV negative MSM. This suggests that a high proportion of MSM and transgender women in Pakistan are at substantial risk for developing anal cancer, in particular those with a concurrent HIV infection. Of note, we found no HSIL cases among HIV-negative participants, possibly partly because they were relatively younger than MSM living with HIV among our participants, but similar to previous findings in an older HIV negative cohort with a mean age of 45 [21]. Although only HSIL is considered to be a precursor of anal cancer, previous studies have shown that about 40% of HIV negative homo- or bisexual men with LSIL at baseline progress to HSIL within only 2–4 years [21, 48]. Moreover, lesions diagnosed as ASC-US may also hide squamous intraepithelial lesions [34, 49], and it is recommended that all individuals with ASC-US or worse on cytology undergo an accurate diagnostic evaluation through HRA guided biopsy as it has high predictive value for Anal Intraepithelial Neoplasia (AIN) [34, 48, 50]. Moreover, one third of ASC-US and LSIL reports are associated with biopsy-diagnosed *high-grade AIN* [50]. Furthermore, it has been reported that AIN 2 and AIN 3 occurs in about 20% of patients with a negative cytology at baseline after an average of 2 years follow-up [34, 51–53].

Concurrent infection with more than one high-risk HPV type has been associated with the presence of [28, 54, 55] or progression to AIN [24, 56]. The earlier work of Palefsky et al. has linked having more than one HPV type to a higher risk of progression [48] or incidence [57] of anal squamous intraepithelial lesions. Similarly, a previous study on MSM living with HIV reported a double risk of ASIL when three or more HPV types were present compared to infection with only one type of HPV [58].

MSM living with HIV had a significantly higher prevalence of high-risk HPV as well as multiple HPV types, both low-and high-risk, compared to the HIV negative participants. A possible explanation is HIV-induced immunosuppression that results in a loss of HPV specific immunity, and reactivation or upregulation of a preexisting HPV infection [58, 59]. A larger number of HPV high-risk types among men living with HIV may also indicate an inability to clear HPV infection [60]. A higher risk of reinfection may also be a factor [61], as seen in our study where MSM living with HIV reported both more unprotected anal intercourse and receptive anal sex preference compared to their counterparts who were HIV negative.

Since infection with any HR-HPV type was associated with threefold increase in risk of having any abnormal anal cytology, it would be important to prospectively monitor MSM and transgender women with high-risk HPV infections and/or HPV/HIV coinfection to prevent invasive lesions and intervene on time to prevent anal cancer, but also to learn more about the factors that influence disease progression. We do know that infection with HPV16 or HPV18 significantly increases the risk of progression to high-grade lesions [55, 56], and that concurrent STIs such as gonorrhea and trichomonas also significantly increase the risk of ASIL [60, 62, 63].

Other studies have indicated receptive anal intercourse as a risk factor for anal lesions [62, 64, 65], but anal HPV infection is not limited to men who have receptive sex [66–68]; it can also be acquired during non-receptive sexual activity. In the current study, abnormal anal cytology was equally prevalent among MSM regardless of anal sex practice, receptive or non-receptive.

This study has some limitations. Firstly, due to lack of equipment and clinicians trained in performing HRA in Pakistan, our analysis of ASIL was based on anal cytology and not on high-resolution anoscopy-driven biopsies, the gold standard for determining disease severity. Awaiting the dispersion of affordable advanced technology, liquid-based cytology, though less sensitive, can be used as a screening tool in high-risk populations in resource-constrained settings as it is convenient, less invasive, cheaper, less time-consuming and requires less expertise than HRA [69]. The fact of the matter is that, recent development in the field of anal cancer screening research has highlighted the potential role for anal Pap testing in providing long-term risk stratification for anal precursors in HIV positive MSM [70]^.^ The sensitivity of anal cytology may also be improved by targeted repeated testing every 12 months among MSM who are HIV-positive but do not yet have signs of anal squamous intraepithelial lesions, or every 2–3 years for HIV-negative MSM [34, 71–73].

A second study limitation is the cross-sectional design, undermining the evaluation of a causal relationship between HPV and abnormal anal cytology, as well as the impact of, i.e., concurrent STI. Thirdly, selection bias may have been introduced by the recruitment of people living with HIV from an ART center while the others came from a community-based organization working for MSM. However, the ART center is the only large center located in Karachi and it represents all ethnic groups in Pakistan. Moreover, given the cultural and religious barriers related to homosexuality and transgender in Pakistan, recruiting our study subjects from an CBO was the only feasible option.

## Conclusion

To the best of our knowledge this is the first study from Pakistan reporting anal HPV associated anal dysplasia in at-risk MSM and transgender women. One third had abnormal anal cytology, and more than half were infected with high-risk HPV. Our study findings indicate the pressing need for further large-scale studies to document the efficacy of anal screening and to develop national guidelines for management of MSM in general and MSM living with HIV in particular. In the absence of the proper infrastructure required for HRA, regular screening with anal cytology of MSM and transgender women may be considered as an initial step in national HIV care and treatment program. Moreover, the benefit and harm analysis of such an approach can only be explored through further screening trials.

## Supplementary Information

**Additional file 1**: **Table S1**. Distribution of HPV types among any Anal Squamous Intraepithelial Lesions (ASIL): results of a study conducted in Karachi Pakistan on MSM and Transgender living with and without HIV infected and uninfected (N= 271).

## Data Availability

The data that support the findings of this study are available from Global and Sexual Health Research Group of Department of Global Public Health on reasonable request and with permission of Karolinska Institutet (KI) Stockholm Sweden. Dr Muslima Ejaz can be contacted on the following email addresses; Muslima.ejaz@aku.edu. Muslima.ejaz@ki.se.
