# Supplementary material for: Human papillomavirus-associated anal squamous intraepithelial lesions in men who have sex with men and transgender women living with and without HIV in Karachi Pakistan: implications for screening and prevention
Source: BMC Infect Dis. 2021 Nov 17;21:1163. doi: 10.1186/s12879-021-06850-w (PMC8597180; doi:10.1186/s12879-021-06850-w)
Supplement: Supplementary file 1 — Additional file 1: Table S1. Distribution of HPV types among any Anal Squamous Intraepithelial Lesions (ASIL): results of a study conducted in Karachi Pakistan on MSM and Transgender living with and without HIV infected and uninfected (N= 271). [file 12879_2021_6850_MOESM1_ESM.docx]

| **Distribution of HPV types among any Anal Squamous Intraepithelial Lesions (ASIL): results of a study conducted in Karachi Pakistan on MSM and Transgender living with and without HIV infected and uninfected (N= 271).** | | | |
| --- | --- | --- | --- |
| **HPV TYPES** | **ASIL positive (n=93)** | **ASIL negative (n=178)** | **ALL N = (271)** |
|  | **N (%)** | **N (%)** | **N** (**%**) |
|  | 93 (35.0) | 178 (65.0) | 271 (100) |
| **HIGH RISK**  **TYPES** |  |  |  |
| HPV 16 | 33 (35.5) ** | 34 (14.8) | 67 (23.2) |
| HPV 18 | 14 (15.1) | 24 (12.7) | 38 (14.0) |
| HPV 31 | 09 (9.5) * | 04 (2.3) | 13 (4.8) |
| HPV 33 | 05 (5.3) | 07 (4.0) | 12 (4.4) |
| HPV 35 | 15 (16.1) * | 26 (11.4) | 41 (15.1) |
| HPV 45 | 09 (9.5) * | 05 (2.8) | 14 (5.2) |
| HPV 52 | 09 (9.5) * | 05 (2.8) | 14 (5.2) |
| HPV 58 | 07 (7.4) | 07 (4.0) | 14 (5.2) |
| HPV 59 | 12 (12.6) * | 08 (4.5) | 20 (7.4) |
| HPV 56 | 06 (6.3) | 08 (4.5) | 14 (5.2) |
| **LOW RISK TYPES** |  | | |
| HPV 6/11 | 43 (45.3) ** | 40 (22.7) | 83 (30.6) |

*P-value 0.01 ** p-value <0.001
